# How rapid is rapid? Exemplary results of real-life rapid rule-out troponin timing in troponin-positive acute coronary syndromes without persistent ST-segment elevation in two contrasting German chest pain unit facilities

**DOI:** 10.1186/s40001-016-0206-0

**Published:** 2016-03-17

**Authors:** Dieter Fischer, Friederike Remberg, Dirk Böse, Michael Lichtenberg, Philipp Kümpers, Pia Lebiedz, Hermann-Joseph Pavenstädt, Johannes Waltenberger, Frank Breuckmann

**Affiliations:** Department of Cardiology and Angiology, University Hospital Münster, Albert-Schweitzer-Campus 1, A1, 48149 Münster, Germany; Department of Cardiology, Arnsberg Medical Center, Arnsberg, Germany; Department of Angiology, Arnsberg Medical Center, Arnsberg, Germany; Department of General Internal Medicine, Nephrology and Rheumatology, University Hospital Münster, Münster, Germany

**Keywords:** Chest pain unit, NSTEMI, Cardiac troponin, Timing, Coronary angiography

## Abstract

**Aim:**

To analyse the timing of cardiac troponin (cTn) measurements in high-risk and cTn-positive acute coronary syndromes without persistent ST-segment elevation (NSTE-ACS) in two structurally different German chest pain units (CPUs), contrasting an urban university maximum care and a rural regional primary care facility.

**Methods:**

All patients encoded as NSTEMI during the year 2013 were retrospectively enrolled in two centres: site (I)—centre of maximum care in an urban university setting and site (II)—centre of primary care in a rural regional care setting. Data acquisition included time intervals from admission to baseline cTn and first and second cTn control as well as type and timing of invasive management.

**Results:**

The median times (site I vs. site II) from admission to cTn result announcement were 26.5 vs. 33.0 min (*p* *=* 0.02) for baseline, 4 vs. 4 h (*p* *=* 0.43) for the first and 11.0 vs. 16.5 h (*p* *=* 0.03) for the second control. Timely announcement, as recommended by guidelines, was available in 86.9 % at baseline, 59.4 % for the first or 41.1 % for the second cTn control. Rates and timing of invasive management were independent from the time point of positive cTn announcement (*p* *=* 0.51 and *p* *=* 0.68, respectively).

**Conclusions:**

German CPUs provide timely identification of cTn-positive patients in a narrow and guideline-adherent time frame using a rapid rule-out protocol. Especially, baseline and early cTn timing was comparable between the urban university maximum care and the rural regional primary care facility without relevant impact on guideline-conforming invasive management, underlining the high standard of care in those highly professional institutions.

## Background

Elevation of cardiac troponin (cTn) directly affects adequate timing of invasive management in patients with acute coronary syndromes without persistent ST-segment elevation (NSTE-ACS), distinguishing between myocardial infarction (MI) without persistent ST-segment elevation (NSTEMI) and unstable angina pectoris (UAP) [1]. Early identification of NSTEMI patients ensures rapid decision-making and initiation of coronary angiography (CA) and has been shown to improve clinical outcome [2–4]. The diagnostic cut-off is defined as a cTn measurement exceeding the 99th percentile of a normal reference population using an assay with a coefficient of variation of ≤10 % at the upper reference limit [5–7]. Whereas conventional cTn testing resulted in cTn-negative or cTn-positive patients, the use of sensitive contemporary or highly sensitive cTn requires further detection and interpretation of a rise or fall in the cTn levels over time [8]. Different protocols indicating correct timing of cTn measurement have been evaluated. As far as the timing is concerned, as to the current guidelines, the high sensitivity of modern tests allows for a rapid rule-out protocol including cTn measurements at baseline and 3 h after admission [3, 9, 10]. Besides a rapid rule-out regimen, for the time of patient inclusion (2013) the German Cardiac Society (GCS) also proposed laboratory measurement after 6–12 h [11]. As far as dynamic changes of cTn levels are concerned, for both, sensitive contemporary and highly sensitive cTn, differences of the relative delta values identifying NSTEMI patients have been implemented.

Following a broad offensive of the GCS, specialized chest pain units (CPUs) have been developed and, now, are thought to ensure quality-of-care in patients with acute coronary syndrome (ACS), providing dedicated standard operating procedures (SOPs) for prompt identification and treatment of patients with an ischaemic aetiology of chest pain, especially for those with acute MI [12]. Individual SOPs are obliged to fulfil current guidelines including timing and interpretation of cTn measurements [9, 11]. However, as to recent registry data, guideline-conforming management still needs to be strengthened even in these highly professional units [13, 14].

The current study aimed to determine the implementation of standardized cTn measurement protocols in two exemplary German CPUs, thereby focusing on real-life timing of cTn sample collection. Moreover, the study aimed to disclose possible differences in a board-certified urban university maximum care versus non-certified rural regional primary care setting.

## Methods

### Study design

Consecutive all-comers with the final diagnosis “NSTEMI” (I21.4) according to the ICD-10-codes admitted during the year 2013 were retrospectively enrolled. After standardized evaluation of the documentation for consistency and completeness by two observers (F.R., F.B.), only patients with clinical signs suggestive of ACS according to the European Society of Cardiology (ESC) guidelines with or without electrocardiographic changes indicative of ischaemia, but without ST-segment elevation, were finally included [1]. Patients with originally unstable angina pectoris reflected by initial cTn above the lower limit without rise or fall of ≤20 % within the controls or by initial cTn below the lower limit control without rise of ≤20 % were excluded.

In general, enrolment was performed in two different sites: site (I)—a centre of maximum care in an urban university setting (University Hospital Münster) and site (II)—a centre of primary care in a rural regional care setting (Arnsberg Medical Centre). In 2013, the department of cardiology of centre I took care of more than 6000 patients in an in-patient and more than 10,000 patients in an out-patient setting, whereas the corresponding department of centre II provided in-patient medical care in over 5000 patients and out-patient care in over 4000 patients. Both sites provide dedicated CPU pathways ensuring rapid and focused assessment of patients with acute thoracic pain based on the local recommendations of the GCS. Only centre I has been board-certified by the GCS [11].

### Troponin measurement and resulting subgroups

#### Patients were subclassified according to different dynamics of cTn levels over time and due to different tests used at the two sites

Site I used a sensitive contemporary assay (ADVIA Centaur TnI-Ultra, Siemens Healthcare) [15, 16]. The values of ≤0.04 ng/ml were judged as negative and values ≥0.05 ng/ml were judged as positive. For this centre, entities were defined as follows: (1) NSTEMI—initial cTn above 0.04 ng/ml, control with a rise or fall of >20 %, (2) NSTEMI—initial cTn below 0.04 ng/ml, control with a rise of >20 %, (3) high-risk (hr)-NSTE-ACS—initial cTn below 0.04 ng/ml, no further control because of prior invasive regimen and (4) hr-NSTE-ACS—initial cTn above 0.04 ng/ml, no further control because of prior invasive management.

Site II used a high-sensitivity assay (Troponin T hs, Roche Diagnostics) [17, 18]. For this assay, the values of <0.014 ng/ml were judged as negative, values between 0.014 ng/ml and 0.05 ng/ml were judged as an observation zone and values >0.05 ng/ml were judged as positive. Except for the additional group (b) [NSTEMI—initial cTn within the observation zone, control with a rise or fall of >50 %], entities were defined analogue to centre I).

### Timing of troponin measurement and coronary angiography

For cTn measurements, times were assessed from admission until the announcement of results according to the electronic documentation, accordingly including laboratory turn-around times. Guideline adherence was defined as follows: admission till announcement of baseline results: <1 h (45–60 min laboratory turn-around time); admission till announcement of the results of the first control: <4 h (control after 3 h plus 1 h laboratory turn-around time) and admission till announcement of the results of the second control <13 h (control after 6–12 h plus 1 h laboratory turn-around time).

Timing of CA was assessed for the time interval from admission to the beginning of CA (puncture times). Guideline-adherent timing of CA was defined as puncture within the first 24 h after admission [1].

### Statistics

Descriptive statistics are based on the available cases. The median with lower and upper quartiles was used for continuous variables without standard deviation. Categorical variables were described by absolute frequencies and percentages. For two independent variables, the Mann–Whitney U test was used. Categorical variables were tested using the Fisher exact test. *p* values ≤0.05 were considered significant without adjustment for multiple testing. Statistical computations were performed using SPSS for Windows (version 22.0).

## Ethical considerations

The aforementioned protocol was reviewed by the local Ethics Committee (i.e. Ärztekammer Westfalen-Lippe and University of Münster, Germany). Given that we only used anonymized clinical routine data from the patients’ hospital records without additional examinations, informed consent by the patients is not required according to § 6 (2) “Gesundheitsdatenschutzgesetz” (health data protection act) North Rhine-Westphalia, Germany. Therefore, the Ethics Committee considers the application and issue of an ethical approval as not necessary.

## Results

A total of 88 patients in site I and 76 patients in site II fulfilled the inclusion criteria. Absolute numbers per group were as follows (site I vs. site II): groups a–c (NSTEMI)—42 vs. 54 patients and groups d–e (hr-NSTE-ACS)—42 vs. 22 patients.

Valid cTn measurements were available in all patients at baseline and at the time of first control unless hr-NSTE-ACS with prior CA. Results of a second control were available in 35 %, adjusted for hr-NSTE-ACS patients with CA prior to second control and consistent diagnosis of NSTEMI after the first control in 92 %.

**Fig. 1:**
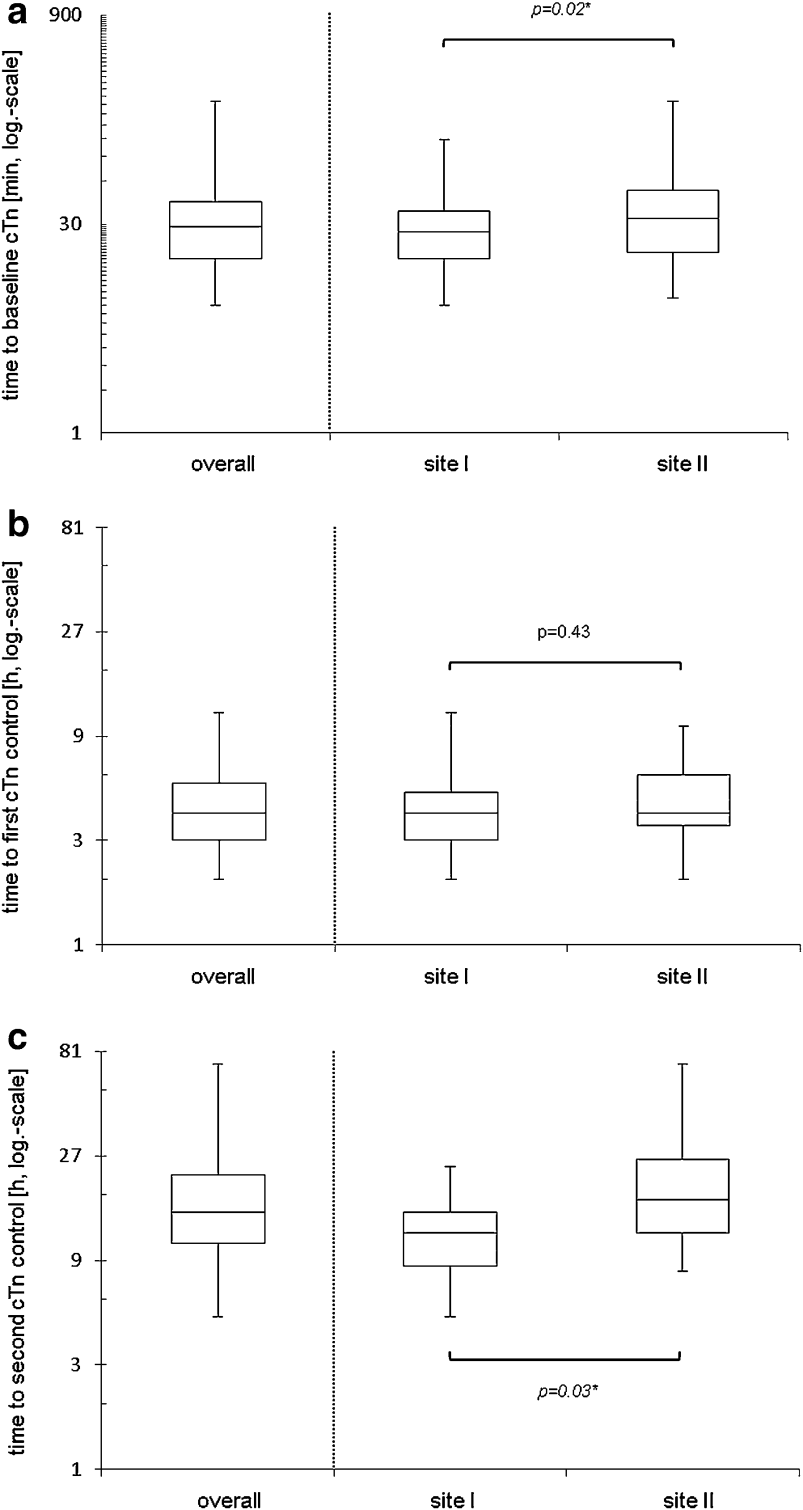
*Boxplot* diagrams visualizing differences of time intervals from admission to results at baseline **a**, first **b** and second **c** cTn control. site I—centre of maximum care in an urban university setting (University Hospital Münster); site II—centre of primary care in a rural regional setting (Arnsberg Medical Center), *asterisk* indicates statistically significance. *cTn* cardiac troponin

Results from baseline cTn were available after a median time of 29 min (17–43 min) with significantly lower time intervals (site I, 26.5 min (17–37 min) vs. site II, 33 min (19–52 min); *p =* 0.02; Fig. 1a) associated with a significantly higher percentage of cTn result announcements in time in centre I. The median time intervals from admission to result of the first and second control were 4 h (4–6 h) and 14 h (10–21 h), respectively. Whereas there was no significant difference concerning the first control (site I, 4 h (3–5 h) vs. site II, 4 h (3.5–6 h); *p =* 0.43; Fig. 1b), time intervals of the second control again differed significantly between both centres (site I, 11 h (8–14 h) vs. site II, 16.5 h (10–22 h); *p =* 0.03; Fig. 1c). When analysing percentages of guideline-adherent timing of cTn measurements, there was no significant difference at the time of first and second control. A detailed subgroup analysis of time intervals and guideline adherence in timing is shown in Tables 1, 2.

**Table 1:** Turn-around times of cTn samples at baseline and after the first and second control in favour of faster results in centre I

Time until result	Overall	Site I	Site II	*p* value
Baseline (min)				
Group (a)	31.0 (19.0–49.0)	26.0 (18.0–39.0)	38.5 (19.0–61.0)	0.05*
Group (b)	20.0 (14.0–46.0)	NA	20.0 (14.0–46.0)	NA
Group (c)	21.0 (14.0–39.0)	17.0 (14.0–28.0)	41.0 (29.0–52.0)	0.05*
Group (d)	27.0 (22.0–34.0)	28.5 (22.0–41.0)	18.0 (18.0–34.0)	0.19
Group (e)	22.0 (19.0–37.0)	31.0 (17.5–37.0)	22.0 (19.0–37.0)	0.74
1st control (h)				
Group (a)	4.0 (3.0–5.0)	4.0 (3.0–5.0)	4.0 (3.0–5.0)	0.95
Group (b)	4.0 (4.0–4.5)	NA	4.0 (4.0–4.5)	NA
Group (c)	4.0 (3.5–6.0)	4.0 (3.0–5.0)	5.75 (3.5–9.0)	0.18
2nd control (h)				
Group (a)	16.0 (11.0–22.0)	13.0 (8.5–16.0)	18.0 (12.0–26.0)	0.08
Group (b)	17.5 (10.0–28.0)	NA	17.5 (10.0–28.0)	NA
Group (c)	14.0 (10.5–16.0)	12.0 (7.0–14.0)	16.0 (14.0–16.0)	0.14

site I: centre of maximum care in an urban university setting (University Hospital Münster); site II: centre of primary care in a rural regional setting (Arnsberg Medical Center)

subgroups: *a–c* myocardial infarction without persistent ST-segment elevation (NSTEMI), *d–e* acute coronary syndromes without persistent ST-segment elevation (NSTE-ACS), *cTn* cardiac troponin

* Statistically significant

**Table 2:** Percentage of cTn result announcement in time of baseline measurement and first and second control

Percentage of cTN in time^a^	Overall (%)	Site I (%)	Site II (%)	*p* value
Moment of cTn measurement				
Baseline	86.9	94.0	78.9	<0.01*
1st control	59.4	61.9	57.4	0.93
2nd control	41.1	52.6	35.1	0.74

*site I* centre of maximum care in an urban university setting (University Hospital Münster); *site II* centre of primary care in a rural regional setting (Arnsberg Medical Center)

^a^defined as admission till announcement of baseline results: <60 min (45–60 min laboratory turn-around time); admission till announcement of results of the 1st control: <4 h (control after 3 h + 1 h laboratory turn-around time); admission till announcement of results of the 2nd control: <13 h (control after 6–12 h + 1 h laboratory turn-around time); *cTn* cardiac troponin

* Statistically significant

Analysis of CA timing within the NSTEMI subset disclosed neither any significant difference between the two sites (*p =* 0.55) nor between diagnoses after the first or second cTn control (*p =* 0.68; Table 3). Guideline-adherent timing of CA could be achieved in 59.1 % (site I, 52.5 % vs. site II, 64.6 %; *p* *=* 0.76; Table 4) in those NSTEMI patients with final diagnosis after the first control. Later diagnosis of NSTEMI due to relevant cTn dynamics not until the second control did not lead to lower rates of adequate CA timing (59.1 % vs. 62.5 %; *p* *=* 0.71; Table 4).

**Table 3:** Timing of CA contrasting NSTEMI patients with final diagnosis after the first versus the second cTn control

Time of CA	Overall	Site I	Site II	*p* value
NSTEMI ensured by the 1st control (h)				
Overall	20.5 h (11.6–45.3 h)	22.0 h (12.3–46.5 h)	20.0 h (10.8–43.5 h)	0.55
Group (a)	22.0 h (11.0–49.8 h)	23.5 h (12.1–51.1 h)	20.0 h (10.0–40.5 h)	0.35
Group (b)	15.0 h (7.0–53.8 h)	NA	15.0 h (7.0–53.8 h)	NA
Group (c)	21.0 h (13.5–35.0 h)	21.0 h (13.0–30.0 h)	21.3 h (15.0–63.9 h)	0.75
NSTEMI ensured by the 2nd control (h)				
Overall	22.3 h (20.5–28.9)	10.0 h; 27.0 h	22.3 h (20.5–28.9 h)	Omitted to due remaining small sample size
Group (a)	20.0 h (15.5–22.0 h)	10.0 h	21.0 h (18.9–38.5 h)	
Group (b)	22.5 h; 31.0 h	NA	22.5 h; 31.0 h	
Group (c)	–	27.0 h	–	

*site I* centre of maximum care in an urban university setting (University Hospital Münster); *site II* centre of primary care in a rural regional setting (Arnsberg Medical Center)

subgroups: *a–c* myocardial infarction without persistent ST-segment elevation (NSTEMI), *CA* coronary angiography, *cTn* cardiac troponin

* Statistically significant

**Table 4:** Guideline-adherent CA timing within 24 h contrasting NSTEMI patients with final diagnosis after the first versus the second cTn control

Percentage of CA in time	Overall (%)	Site I (%)	Site II (%)	*p* value
NSTEMI ensured by the 1st control (h)				
Overall	59.1	52.5	64.6	0.76
Group (a)	57.7	50.0	65.4	0.81
Group (b)	62.5	NA	62.5	NA
Group (c)	60.0	57.1	66.7	0.90
NSTEMI ensured by the 2nd control (h)				
Overall	62.5	50.0	66.7	Omitted to due remaining small sample size
Group (a)	66.7	50.0	75.0	
Group (b)	50.0	NA	50.0	
Group (c)	–	–	NA	

*site I* centre of maximum care in an urban university setting (University Hospital Münster); *site II* centre of primary care in a rural regional setting (Arnsberg Medical Center)

subgroups: *a–c* myocardial infarction without persistent ST-segment elevation (NSTEMI), *CA* coronary angiography, *cTn* cardiac troponin

* Statistically significant

Including both NSTEMI and hr-NSTE-ACS patients, an invasive strategy was chosen in 98.8 % of patients (site I, 98.8 % vs. site II, 98.7 %, *p* *=* 0.65). Rates of percutaneous coronary intervention (PCI) were 64.3 % (site I) and 61.8 % (site II, *p =* 0.90), respectively. Coronary artery bypass graft (CABG) placement was significantly more often performed in site II (site I, 3.6 % vs. site II, 16.7 %, *p* *=* 0.02). For NSTEMI only, rates of intervention were nearly similar in cTn-positive patients ensured by the first or second control (71.6 vs. 87.5 %; *p* *=* 0.51) and between both sites (site I, 70.0 % vs. site II, 72.9 %; *p* *=* 0.82, Table 5). CABG procedures were more often performed in site II (site I, 2.5 % vs. site II, 16.7 % for NSTEMI ensured by first control, *p* < 0.01; site I, 0 vs. 16.7 % for NSTEMI ensured by the second control).

**Table 5:** Rates of intervention including PCI and CABG in NSTEMI patients with final diagnosis after the first versus the second cTn control

Rates of intervention	Overall	Site I (%)	Site II (%)	*p* value
NSTEMI ensured by the 1st control (h)				
Overall	71.6	70.0	72.9	0.82
PCI	61.4	67.5	56.3	0.73
CABG	10.2	2.5	16.7	<0.01*
None	28.4	30.0	27.1	0.82
NSTEMI ensured by the 2nd control (h)				
Overall	87.5	100	83.3	Omitted to due remaining small sample size
PCI	75.0	100	66.7	
CABG	16.7	–	16.7	
None	16.7	–	16.7	

*site I* centre of maximum care in an urban university setting (University Hospital Münster); *site II* centre of primary care in a rural regional setting (Arnsberg Medical Center)

subgroups: *a–c* myocardial infarction without persistent ST-segment elevation (NSTEMI), *CA* coronary angiography, *cTn* cardiac troponin

* Statistically significant

## Discussion

The implementation of specialized CPUs has been shown to improve the prognosis of patients with ACS [19, 20]. These units allow a prompt identification and treatment of patients with suspected ACS by use of standardized diagnostic and therapeutic protocols [21]. Contemporary, more sensitive cTn assays allow a more rapid and more precise diagnosis of NSTE-ACS patients [22, 23]. The current guidelines recommend a rapid rule-out protocol within three hours after presentation using high-sensitive cTn assays [1]. Our study analysed the “real-life” situation for the timing of the diagnosis and invasive approach for NSTEMI patients in two German centres, a centre of maximum care in an urban university setting and a centre of primary care in a rural regional care setting. Our study carries the following key messages: (1) in about 90 % of patients the announcement of baseline cTn results was possible within one hour as requested by guidelines; (2) the results of the first cTn control were available in a guideline-adherent median time frame of about four hours; however, result announcement was still considered as too late in about 40 % of the cases; (3) a second cTn control was necessary in roughly one-third of the patients only; nonetheless, result announcement was too late in nearly half of the patients included; (4) there was a significant difference between the two contrasting exemplary sites at baseline and the second control in favour of faster announcement of cTn results within the university setting; and (5) most remarkably, the different timelines for the announcement of troponin levels between both sites, however, had no impact on the guideline-conforming timing of an invasive approach.

So far, only little has been known about guideline adherence in NSTEMI patients within the CPU concept [24, 25]. Previous data from the CPU registry on the one hand demonstrated that cTn-positive CPU patients were faster and more often treated with PCI than cTn-negative patients, but on the other hand that there was also a low adherence to the standard of care as proposed by the ESC guidelines [25]. Rapid rule-out cTn protocols are thought to ensure early and facilitated decision-making associated with more guideline-conforming initiation of CA [3, 26]. However, as to our knowledge, validation and analysis of the real speed of cTn result announcement within the CPU is missing. In our exemplary study, there was a significant difference of baseline cTn result announcement in favour of the university institution, which may be due to stronger SOPs in that CPU and/or more human and technical resources in the corresponding laboratory, especially at night or on weekends [27, 28]. Nonetheless, times were adequate and mostly below 60 min in both sites without a relevant disadvantage of the non-certified rural regional primary care facility. For the first troponin control, the guideline-conforming timelines were reached less frequently than the baseline measurements with a further degradation as far as the second control is concerned. Whereas for the first control measurements no significant difference was seen for both sites, the maximum care university facility provided faster results of the second control, pointing towards an assumed higher awareness of the benefit of repetitive cTn measurements in site I, again related to a potentially higher degree of education of the CPU personal in that maximum care setting also ensuring certified SOPs [9, 11, 14]. In contrast, one still has to reflect that even in that highly trained facility timely cTn was available in about two-thirds (first control) or even worse a half (second control) only, allowing for further structural improvement and thereby underlining the necessity for such benchmarking processes [29].

The continuous development of increasingly sensitive cTn tests provides progressively shorter time intervals until the diagnosis of an NSTEMI may be confirmed. So far, even 2-hour protocols have been introduced [30, 31]. Besides this optimism, one should simultaneously keep in mind that this development particularly is important for those patients with a low to intermediate pretest probability without relevant risk markers for an early invasive procedure, allowing for early and safe discharge as the gatekeeper for further in-hospital work-up [32]. The question remains whether the implementation of those rapid protocols also favours a better supply in cTn-positive patients with regard to an earlier initiation of an invasive regimen [33, 34]. Thus, the timely diagnosis and treatment of patients with NSTE-ACS, in contrast to patients presenting with STEMI, remains challenging [35]. For example, the optimal timing for the invasive procedure remains uncertain. Recently, some randomized trials have investigated the timing of intervention in NSTE-ACS patients. Based on these trials, current ESC guidelines recommend an immediate invasive strategy for hr-NSTE-ACS patients within two hours, an early invasive strategy within 24 h for patients with high-risk features defined by a GRACE score > 140 and a primary high-risk criterion and within 72 h for those at lower risk [1]. A more recent trial (LIPSIA-NSTEMI) randomized “stable” patients with NSTEMI in an immediate, early or selective invasive approach [36]. The authors concluded that in NSTEMI patients an immediate invasive approach does not offer an advantage over an early or a selective invasive approach with respect to large myocardial infarctions as defined by peak CK-MB levels, which is also supported by similar clinical outcomes. According to our data, even though there was a significant difference in the frequency of recommended bypass graft surgery which may be due to the assumption that university hospitals provide superior background and frequency in performing complex interventions, in general, rates of interventions were high, comparable between both sites, independent from the accuracy of the rapid rule-out protocol and the time of first relevant cTn delta as well as comparable to larger recent trials (i.e. ACUITY and PLATO) [37–39].

## Limitations

The main limitation of this study is the small sample size that may bias the results. The inclusion of only two sites may not be considered representative for the quality-of-care evaluation in the entire German CPUs. Second, a clinical follow-up for this retrospective analysis was not performed, so that the impact of guideline-conforming diagnosis and therapy on further cardiovascular events remains unsolved for this cohort. Third, the use of different cTn assays may have biased the results, especially as group (b) was restricted to the high-sensitivity assay of site II, only.

## Conclusion

After the first years of implementation, our exemplary data indicate that German CPUs provide timely identification of cTn-positive patients in a narrow and guideline-adherent time frame. Whereas baseline and early cTn timing using a rapid rule-out protocol appears to be comparable between rural regional primary and urban university maximum care facilities, frequency and timing of a potential second cTn control is superior in the later. Differences in cTn timing did not directly affect type and initiation of guideline-conforming CA. However, its impact on hard end points in terms of cardiovascular events during index stay and follow-up still needs to be determined.
